# Utility of the Physical Examination in Detecting Pulmonary Hypertension. A Mixed Methods Study

**DOI:** 10.1371/journal.pone.0108499

**Published:** 2014-10-24

**Authors:** Rebecca Colman, Heather Whittingham, George Tomlinson, John Granton

**Affiliations:** 1 Pulmonary hypertension program, Toronto General Hospital, Division of Respirology, Faculty of Medicine, University of Toronto, Toronto, Ontario, Canada; 2 Department of Critical Care Medicine, Hamilton General Hospital, McMaster University, Hamilton, Ontario, Canada; 3 Department of Medicine, Mount Sinai Hospital and University Health Network, Faculty of Medicine, University of Toronto, Toronto, Ontario, Canada; VU University Medical Center, Netherlands

## Abstract

**Introduction:**

Patients with pulmonary hypertension (PH) often present with a variety of physical findings reflecting a volume or pressure overloaded right ventricle (RV). However, there is no consensus regarding the diagnostic utility of the physical examination in PH.

**Methods:**

We conducted a systematic review of publications that evaluated the clinical examination and diagnosis of PH using MEDLINE (1946–2013) and EMBASE (1947–2013). We also prospectively evaluated the diagnostic utility of the physical examination findings. Patients who underwent right cardiac catheterization for any reason were recruited. After informed consent, participants were examined by 6 physicians (3 “specialists” and 3 “generalists”) who were unaware of the results of the patient's hemodynamics. Each examiner independently assessed patients for the presence of a RV lift, loud P2, jugular venous distension (JVD), tricuspid insufficiency murmur and right-sided 4th heart sound at rest and during a slow inspiration. A global rating (scale of 1–5) of the likelihood that the patient had pulmonary hypertension was provided by each examiner.

**Results:**

31 articles that assessed the physical examination in PH were included in the final analysis. There was heterogeneity amongst the studies and many did not include control data. The sign most associated with PH in the literature was a loud pulmonic component of the second heart sound (P2). In our prospective study physical examination was performed on 52 subjects (25 met criteria for PH; mPAP ≥25 mmHg). The physical sign with the highest likelihood ratio (LR) was a loud P2 on inspiration with a LR +ve 1.9, 95% CrI [1.2, 3.1] when data from all examiners was analyzed together. Results from the specialist examiners had higher diagnostic utility; a loud P2 on inspiration was associated with a positive LR of 3.2, 95% CrI [1.5, 6.2] and a right sided S4 on inspiration had a LR +ve 4.7, 95% CI [1.0, 15.6]. No aspect of the physical exam, could consistently rule out PH (negative LRs 0.7–1.3).

**Conclusions:**

The presence of a loud P2 or audible right-sided 4th heart sound are associated with PH. However the physical examination is unreliable for determining the presence of PH.

## Introduction

Pulmonary hypertension (PH) is defined as a mean pulmonary artery pressure (mPAP) ≥25 mmHg measured during cardiac catheterization. The World Health Organization Dana Point Classification divides pulmonary hypertension into five groups based upon similarities in therapeutic approaches, and to some extent, pathophysiologic mechanisms [1]. Although the natural history varies according to the etiology of the condition, PH is often a progressive disease characterized by increased pulmonary vascular resistance and diminished right ventricular (RV) function due to increased RV afterload [2].

Early in the disease, the symptoms of PH are often benign and non-specific but progress over time to functionally limiting dyspnea and fatigue. Patients may also experience chest pain, palpitations, pre-syncope, syncope and peripheral edema. The non-specific nature of symptoms in early disease and subtlety of clinical signs are some of the obstacles to establishing an early diagnosis. Delays in the diagnosis of PH lead to postponement of treatment and thus may have deleterious effects.

Patients with pulmonary hypertension are reported to present with a variety of physical findings reflecting a volume and/or pressure overloaded right ventricle (RV). These include a left parasternal lift, an accentuated pulmonary component of the second heart sound (P2), a pansystolic murmur of tricuspid regurgitation (TR), a diastolic murmur of pulmonary insufficiency and a third or fourth heart sound originating in the RV. In more advanced states patients may manifest jugular venous distension, hepatomegaly and peripheral edema [3]. Numerous studies have described the various findings on physical examination of PH patients but there is no consensus regarding their diagnostic utility [2], [4]–[6].

Many early studies used phonocardiography to validate the role of the physical examination in the diagnosis pulmonary hypertension. With increased adoption of 2-dimensional and Doppler echocardiography, the phonocardiogram has fallen into disuse. Attempts to improve diagnostic accuracy of non-invasive tools other than echocardiography have included revisiting the phonocardiogram itself as well as acquisition of similar phonocardiographic data through use of electronic stethoscopes and computerized algorithms [7]–[11]; however the use of such tools is impractical for most clinicians, and most continue to rely on the physical examination itself, even though, to date, the diagnostic utility of the physical examination in determining the presence of PH in a symptomatic patient has not been systematically evaluated.

Our purpose was to evaluate the diagnostic utility of the physical examination in PH through literature review and empirical study. We systematically reviewed and appraised the published literature on the physical examination in PH. In addition we prospectively assessed the diagnostic utility of the various physical signs of PH described in the literature by correlation with results of right heart catheterization (RHC). (A description of physical examination techniques used to evaluate for pulmonary hypertension can be found in the appendix.) We also evaluated the potential impact of differences in observer experience (specialist vs generalist) on the detection of findings on physical examination.

## Methods

### Systematic review

We performed a systematic search of the published literature using MEDLINE (1946–Feb 2013) and EMBASE (1947–March 22, 2013) to identify original publications that evaluated the clinical examination and diagnosis of pulmonary hypertension. We used the following key terms or Medical Subject Headings: (EXP Hypertension, Pulmonary) AND (EXP Physical Examination OR EXP Heart Auscultation OR Heart sounds. The search was limited to English language and human studies. The titles and abstracts of articles retrieved were reviewed by two of the authors (R.C, J.G). If either reviewer chose an article as possibly useful, the article was reviewed for content. Differences between the reviewers regarding articles to be included were resolved by consensus. Reference lists from appropriate articles were carefully searched for other relevant articles. Publications were excluded if they were case reports or review articles.

### Physical examination study

Six academic staff physicians, comprising three “experts” (recruited from the heart failure program (1), the division of respirology (1) and lung transplant program (1) and three “non-experts” (two from the division of general internal medicine), acted as examiners in the study. All examiners were blinded to the patients' diagnoses and none were involved in any aspect of the patients' care. All physicians participated in a common training session in which exam maneuvers were reviewed and subjects who demonstrated the findings of interest were available. Following the training session, the physicians were asked to examine a group of patients for the presence or absence of five pre-specified physical findings: a loud P2, a right ventricular heave, a right-sided S4, a murmur of tricuspid regurgitation and the height of the jugular venous distention (JVD) (Text S1). Examination was performed during quiet active breathing. Each sign was re-evaluated during a slow inspiratory maneuver followed to approximately 75% of total lung capacity to help augment right-sided auscultatory findings. The examiners documented their findings on a standardized recording sheet (Figure S1). After completing the series of maneuvers the examiners were asked to estimate the probability that the patient either did have or did not have PH by indicating the degree of certainty using a Likert scale.

The examinations occurred in a series of private examination rooms where examiners moved from one room to the next and were not permitted to discuss their findings.

### Subject selection

Subjects were prospectively recruited from a list of patients who underwent right heart cardiac catheterization for any indication at a large tertiary care teaching hospital with an active PH program. Subjects were contacted by telephone and invited to participate in the study using a standardized script. Based on results of the right heart catheterization, subjects were divided into two groups: subjects with PH (those with mPAP >25 mmHg) and those with normal pulmonary artery pressures (mPAP ≤25 mmHg). Each subject was advised about the intent of the study and further advised not to discuss any aspect of their health or their diagnosis with the examining physicians. The examiners were unaware of the subjects' hemodynamics or diagnoses. Prior to the study, standardized instruction on the slow inspiratory maneuver was provided to all patients by one of the authors (JG). The study was approved by the research ethics board of the University Health Network (REB number. All participants signed written informed consent before study enrollment.

### Statistical analysis

Published raw data was obtained from relevant systematic review articles whenever available and used to construct 2×2 contingency tables for clinical variables. When data for a specific variable were available from more than one source, these were combined across studies. We then used this published data to calculate sensitivity, specificity and summary positive and negative likelihood ratios (LR) along with 95% confidence intervals (CI).

For each maneuver in our prospective study, we fitted a random effects logistic regression model to account for the fact that six separate examiners assessed each subject and to allow estimation of sensitivity, specificity and the LRs for the average examiner. The probability of a patient having a positive finding on the maneuver performed by a particular examiner was related through a logit link to the patient's true PAH status and a random effect for the examiner. We also fitted a model that allowed for different average sensitivity and specificity for specialists and generalists. The models were fitted using Bayesian methods using the package rjags [12], with three parallel chains run for 10,000 burn-in iterations and 10,000 further iterations after convergence was established using the Gelman-Rubin statistic. Diffuse normal priors were used for regression parameters and uniform prior distributions on the range 0 to 1 were used for the standard deviations of the random effect. Posterior medians and 95% credible intervals (CrI) were extracted from the posterior samples [13]. For each examiner, we used the global rating to generate the non-parametric ROC curve and calculated the area under the curve and its 95% CI [14].

## Results

### Systematic Review

**Figure 1:**
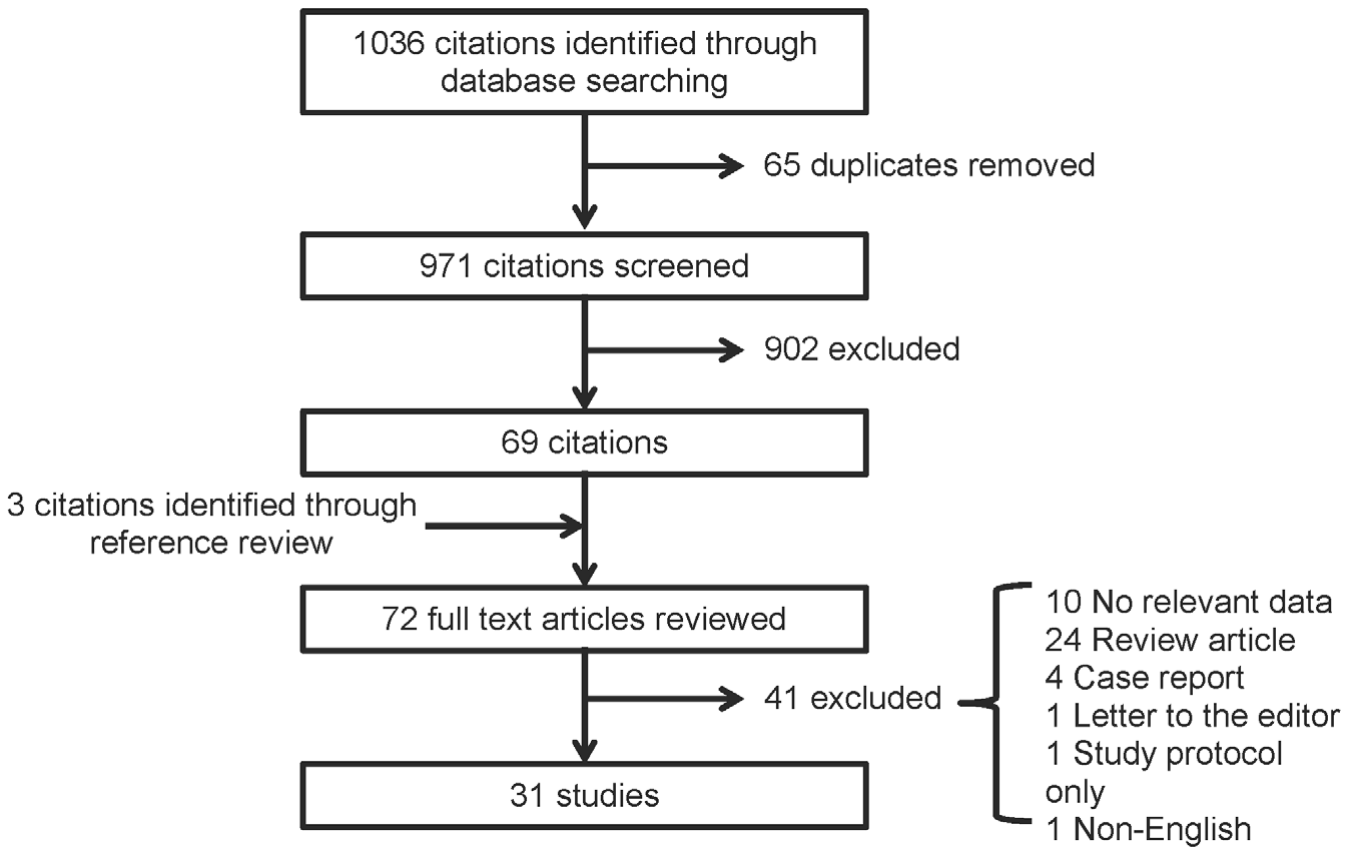
Summary of literature search and identification of publications.

The literature search identified 1036 citations (Figure 1). Of these, 65 were determined to be duplicates and 902 were excluded after review of their titles and abstracts. Review of the references from the 69 remaining studies identified three additional studies for full text evaluation. Thus 72 studies were reviewed in detail. Of these, 31 studies were identified that assess the physical examination in pulmonary hypertension; these studies were included in the final analysis (Table S1).

There was significant heterogeneity among the studies reviewed in regard to study population, subject selection, methods/details of evaluating physical findings and results. Of the 31 studies in patients with PH, 10 [8], assessed splitting of the second heart sound (S2), but results were inconsistent. For example Bleifer *et al.* (1960) and Wood (1952) described that patients with PH exhibit narrow splitting of S2 while Xu et. al (2002) argued that as pulmonary artery pressure increases, so does the A2-P2 interval [8], . Using an intracardiac phonocardiogram at the time of right heart catheterization, Shaver *et al.* (1974) demonstrated that patients with pulmonary hypertension can have varying degrees of splitting of S2 [20].

Because the majority of papers did not include control data, the results from only six studies could be used to determine the performance characteristics of physical examination findings, and only 5 in regard to individual components of the physical examination. Several used phonocardiography to identify the characteristics of the components of the heart sounds. Ungerer *et al.* studied 49 patients with systemic sclerosis and compared the test characteristics of the physical examination, chest radiograph, EKG, echocardiogram, and pulmonary function testing (vital capacity and single breath diffusing capacity. However they defined PAH as a mean PA pressure at rest greater than 20 mmHg or an abnormal increase in PA pressure with exercise [24]. Using this unconventional definition, 16 patients met the criteria for PAH. They reported that the physical examination by two cardiologists (incorporating a prominent a-wave, parasternal heave, an increased S2, and or a right ventricular gallop was positive in 8/16 patients and in 4/33 scleroderma patients in the cohort without PAH providing a sensitivity of 50% [95% CI, 28% to 72%] and a specificity of 88% [95% CI, 73% to 95%] with a corresponding positive LR of 4.1 [95% CI, 1.5–11.7] and negative LR of 0.57 [95% CI, 0.34 to 0.94]. The details of the examination methods and relative merit of one finding over the other were not provided in this high-risk population for PAH.

In 1968, Sutton *et al.* evaluated the second heart sound in patients with pulmonary hypertension in a variety of conditions including congenital cardiac lesions, idiopathic pulmonary hypertension and chronic respiratory disease [25]. They also included a small number of “control” subjects in their analysis: patients with mitral regurgitation or intra-cardiac shunts without PH. In a second paper published in the same journal issue, the authors describe their findings regarding second heart sounds in normal subjects obtained using similar methods [26]. By combining the data from these two papers, the presence of a loud P2 (defined as the pulmonary component of the second heart sound heard louder than the aortic component (A2), assessed where both components can be heard simultaneously during splitting) has a sensitivity of 45.5% [95% CI: 36%–55%] and a specificity of 99% [95% CI: 96%–100%] with a corresponding positive LR of 56.4 [95% CI: 7.9–401.7] and negative LR of 0.5 [95% CI: 0.46–0.66] (Table 1). Another study in our review evaluated clinical predictors of PAH in 55 patients undergoing orthotopic liver transplantation and found that a loud P2 had a sensitivity of 37.5% [95% CI: 14%–69%] and a specificity of 98% [95% CI: 89%–100%] while an RV heave had a sensitivity of 37.5% [95% CI: 14%–69%] and a specificity of 96% [95% CI: 86%–99%](Table 1) [27]. However, only eight patients in this study had pulmonary arterial hypertension (PAH), limiting the precision of the estimates of sensitivity. In 1980 Stein and Sabbah reported intracardiac sound and pressure recordings in 24 subjects in an attempt to determine whether RV failure modified the intensity of P2 in pulmonary hypertension. Eight subjects had pulmonary hypertension with normal RV function, 8 had pulmonary hypertension with evidence of RV dysfunction and 8 were controls with normal PAP [28]. They found that the amplitude of P2 was significantly higher in the pulmonary hypertension subjects compared to controls. There was no significant difference in amplitude of P2 in subjects with PH and evidence of right heart failure compared to those with PH and normal RV function. Because the results of the study are provided as average sound pressure measurements for an entire group, data to assess the performance characteristics of a loud P2 in a given patient are not available in the publication.

**Table 1:** 2×2 contingency tables and derived test characteristics from relevant studies.

Study	Finding	PH	No PH	Sn	Sp	LR+ve	LR −ve
Sutton, Harris & Leatham [25] AND Harris & Sutton [26] (1968)	P2>A2			45.5%	99%	56.36 [7.91–401.72]	0.55 [0.46–0.66]
	Finding +ve	45	1				
	Finding −ve	54	123				
Sutton, Harris & Leatham [25] (1968)	P2>A2			45.5%	98.6%	31.85 [2.02–503.37]	0.55 [0.46–0.66]
	Finding +ve	45	0				
	Finding −ve	54	34				
Sutton, Harris & Leatham [25] (1968)	P2≥A2			72.7%	70.6%	2.47 [1.45–4.22]	0.39 [0.26–0.57]
	Finding +ve	72	10				
	Finding −ve	27	24				
Pilatis [27]. (2000)	‘loud P2’			37.5%	97.9%	17.63 [2.08–149.12]	0.64 [0.37–1.09]
	Finding +ve	3	1				
	Finding −ve	5	46				
Pilatis [27]. (2000)	RV heave			37.5%	95.7%	8.81 [1.74–44.74]	0.65 [0.38–1.12]
	Finding +ve	3	2				
	Finding −ve	5	45				
Ungerer [15]. (1983)	“Physical exam			50%	95.2%	4.12 [1.46–11.68]	0.57 [0.34–0.94]
	Finding +ve	8	4				
	Finding –ve	8	29				

Sn: sensitivity, SP: specificity, LR +ve: positive likelihood ratio, LR−ve: negative likelihood ratio, RV: right ventricle.

Recently, Chan *et al.* investigated the quantitative relationship between acoustic characteristics of S1 and S2 in patients with PAH compared to controls using phonocardiography [7]. They demonstrated that the normal acoustic profile observed in controls, of increased S1 complexity and intensity compared to S2, was altered in subjects with PAH; the PAH subjects were found to have increased S2 complexity compared to S1. Additionally, the mPAP was found to be an independent predictor of S2 complexity in patients with PAH [7]. Similar to the Stein study described above, the results are provided as average sound intensity and complexity measurements for the entire group therefore making it difficult to determine performance characteristics of the loud P2.

### Physical examination study

In our empiric study, physical examination was performed on 52 subjects. Of these, 25 had mPAP >25 mmHg. Baseline characteristics of study participants are provided in Table 2. The two groups were similar with respect to age and sex. There were slightly more patients with a diagnosis of connective tissue disease in the normal PAP group.

**Table 2:** Baseline characteristics of study population.

Variable	Mean PAP ≤25 mmHg (N = 27)	Mean PAP >25 mmHg (N = 25)
Mean/Median PA pressure	**17/16 mmHg**	**35.9/32 mmHg**
Range of PA pressure	**10–25 mmHg**	**26–61 mmHg**
Age (median, range)	64 (33–83)	65.5 (28–82)
Female	10	10
**Diagnosis**		
Mitral valve disease	**6**	**5**
Aortic valve disease	**7**	**0**
Cardiomyopathy	**2**	**6**
Ischaemic heart disease	**0**	**5**
Lung disease	**4**	0
Connective tissue disease	**2**	0
PAH*	**0**	6
Other	**6**	3
**Wedge pressure or left ventricular end-diastolic pressure >15 mmHg**	**0**	**17**

As shown in Table 3, when data from all examiners were analyzed together no sign reliably predicted nor excluded the presence of pulmonary hypertension. While many of the signs had high specificities (88% for an S4 on inspiration, 85% for a loud P2 on inspiration, 84% for an RV lift on inspiration), the sensitivities were low (12%, 29% and 21% respectively). The physical sign with the highest positive LR was a loud P2 on inspiration (LR +ve 1.9, 95% CrI [1.2, 3.1]) and all negative LRs were approximated 1.0.

**Table 3:** Summary of test characteristics for each component of the physical examination that was performed, with comparisons between specialists and generalists.

Sign	All Examiners				Specialists				Generalists			
	Sn	Sp	LR+	LR−	Sn	Sp	LR+	LR−	Sn	Sp	LR+	LR−
**RV Lift**	32	72	1.1 [0.78,1.6]	1.0 [0.81,1.1]	34	73	1.3 [0.74,2.1]	0.9 [0.72,1.1]	31	70	1.0 [0.58,1.7]	1.0 [0.78,1.3]
**RV Lift (insp)**	21	84	1.3 [0.78,2.1]	1.0 [0.82,1.1]	32	76	1.4 [0.79,2.5]	0.9 [0.68,1.1]	13	88	1.2 [0.42,2.8]	1.0 [0.85,1.1]
**Loud P2**	43	74	1.6 [1.2,2.4]	0.8 [0.63,0.92]	52	70	1.8 [1.2,2.8]	0.7 [0.48,0.90]	34	77	1.5 [0.84,2.6]	0.9 [0.67,1.1]
**Loud P2 (insp)**	29	85	1.9 [1.2,3.1]	0.8 [0.71,0.96]	37	87	3.2 [1.5,6.2]	0.7 [0.51,0.90]	22	81	1.2 [0.58,2.4]	1.0 [0.78,1.2]
**S4**	20	83	1.2 [0.73,2.0]	1.0 [0.84,1.1]	16	94	3.0 [0.94,8.3]	0.9 [0.77,1.0]	28	69	0.94 [0.52,1.6]	1.0 [0.82,1.3]
**S4 (insp)**	12	88	1.0 [0.51,1.9]	1.0 [0.91,1.1]	13	96	4.7 [1.0,15.6]	0.9 [0.79,1.0]	13	77	0.6 [0.24,1.2]	1.1 [0.96,1.4]
**TR**	28	69	0.9 [0.62,1.3]	1.0 [0.89,1.2]	30	82	1.8 [0.9,3.2]	0.9 [0.67,1.0]	26	56	0.6 [0.33,0.95]	1.3 [1.0,1.8]
**TR (insp)**	22	81	1.1 [0.71,1.8]	1.0 [0.85,1.1]	22	86	1.7 [0.77,3.4]	0.9 [0.75,1.1]	22	75	0.93 [0.46,1.6]	1.0 [0.86,1.3]

However, when results from examination by the specialist group were analyzed separately from those from the “generalist group”, a loud P2 on inspiration (specificity 87%, sensitivity 37%, positive LR 3.2, 95% CrI [1.5, 6.2]) and a right sided S4 on inspiration (specificity 96%, sensitivity 13%, positive LR 4.7, 95% CrI [1.0, 15.6]) were the best predictors of pulmonary hypertension. The same maneuvers are not predictive when performed by the generalist physicians (positive LR 1.2, 95% CrI [0.58, 2.4] and 0.6, 95% CI [0.24, 1.2] respectively). Although the differences between LRs in specialists and generalists were not large for these two maneuvers, the probabilities that the specialists had larger LRs were 96% and 97%. We found no aspect of the physical exam, alone or used in combination in a global rating, could consistently rule out pulmonary hypertension, with estimates of negative LRs ranging from 0.7 to 1.3. (Table 3).

When examiners were asked to estimate their confidence as to whether or not a patient had pulmonary hypertension at the end of the assessment (presumably based on a gestault of their findings and clinical biases), the accuracy of diagnosis was quite low. The areas under the ROC curves for specialists ranged from 0.57–0.62 while for generalists they ranged from 0.41–0.56 (Table 4).

**Table 4:** ROC curves for each physician's estimate of the probability that the patient either did have or did not have PH.

Examiner	Area under ROC curve (95% C.I.)
Specialist 1	0.61 (0.45–0.77)
Specialist 2	0.57 (0.39–0.75)
Specialist 3	0.62 (0.44–0.80)
Generalist 1	0.43 (0.25–0.61)
Generalist 2	0.56 (0.38–0.74)
Generalist 3	0.41 (0.21–0.61)

The degree of certainty was estimated for each patient at the end of the examination using a Likert scale.

## Discussion

In this study, we sought to validate the physical examination for pulmonary hypertension through review of the literature and empirical study. Although the accurate diagnosis of PH is important, there are very few prior studies evaluating the role of the clinical examination. The results of the available studies are often not applicable to present practice, especially because phonocardiography is no longer generally available. As well, although most of the papers derive from an era in which there was heavier reliance on physical examination for diagnosis that was also an era when less emphasis was placed on study design, especially noted in the absence of control groups. Consequently historical studies have methodological limitations relating to cohort assembly, lack of controls and uncertain blinding. Our review found that the literature is dominated by heterogeneous, small, cross-sectional and retrospective studies. Consequently, it is not possible to form evidence-based conclusions from the existing published data.

A common theme in the limited studies available is investigator interest in the characteristics of the second heart sound in pulmonary hypertension. In that regard, the utility of the duration of the A2 to P2 interval (S2 splitting) for estimation of pulmonary pressure is questionable. In our study, we did not formally evaluate duration of S2 splitting given the inconsistent literature and the difficulty in accurately assessing and documenting the finding with tools available in routine clinical practice. The literature does support that the presence of a loud P2 is associated with an increased likelihood of pulmonary hypertension while its absence may make the diagnosis less likely. However, different authors provide varying definitions of “loud” thus compromising the generalizability of the finding.

The results of our clinical evaluation do support the notion derived from prior studies, limited though they may be, that a loud P2 is a physical sign predictive of pulmonary hypertension. We found, however, that meaningful value was only achieved when the assessment was made by a specialist (with experience in examining patients with PH) during a slow inspiratory maneuver. Even so, in our evaluation, the maximum LR +ve associated with this finding was only 3.2, much lower than the LR+ve of >56 calculated from the data of Sutton *et al.* This difference may be explained by differences in study design and subject selection. The high testing characteristics obtained by Sutton *et al.* are likely an overestimation stemming from a study of a cohort of patients with a known diagnosis of pulmonary hypertension. In addition, they used phonocardiography, rather than auscultation, to evaluate the heart sounds. In our study, we evaluated patients referred for right heart catheterization, without prior knowledge of pulmonary pressures. Nevertheless the population of patients that we studied likely has a higher prevalence of PH than the general population, because they had some indication for measurement of right ventricular/pulmonary hemodynamics. Consequently, our test characteristics may overestimate utility of the manoeuvres that we evaluated if they were applied to the general population. Furthermore the physical examination, used because we felt that our findings would be more applicable to everyday practice, is likely to be less precise than phonocardiography in assessing the absolute and relative loudness of heart sounds. This may also explain why in contrast to the literature the absence of a loud P2 cannot not be used to rule out PH.

In our study the finding that was most predictive of PH (LR +ve 4.7) was a right-sided S4. There are claims in the literature that the presence of a right-sided S3 or S4 supports PH but published data in the literature to support such claims is lacking.

In our study, “specialists” performed slightly better than “generalists”. This is not surprising when one considers the subtlety of the positive findings. An individual with a trained ear would presumably have less difficulty recognizing a loud P2 compared to someone with less experience. Similarly, a clinician who is unaccustomed to assessing for right-sided heart sounds may not easily appreciate sounds that are louder, or only noticeable, on inspiration, such as a right-sided S4.

Consistent with published data, we found that physical examination is more reliable at ruling disease in, based on the higher specificity and positive likelihood ratios. In contrast, low sensitivity and negative likelihood ratios close to 1.0 preclude reliance on negative findings on physical examination to exclude presence of pulmonary hypertension.

In conclusion, although we demonstrate that the physical findings of a right-sided S4 and a loud P2 are associated with PH, the physical examination is unreliable overall for the diagnosis of PH. Positive findings are only modestly predictive when their presence is noted by “specialists” and they are not predictive when noted by “generalists”. Absence of findings is of no use to exclude the diagnosis of PH. Previously published literature manifests weaknesses in subject selection and study design that has led to overestimation of the utility of physical examination findings to support or exclude the diagnosis of pulmonary hypertension.

## Supporting Information

Figure S1 **Standardized recording sheet used by examiners to record their findings for each patient.** A Likert scale was used to estimate the examiner's impression regarding the likelihood of pulmonary hypertension at the end of each examination.
(TIFF)

Table S1 **Summary of the 31 studies were identified that assess the physical examination in pulmonary hypertension and were included in the final analysis.**
(DOCX)

Text S1 **Details of the physical examination maneuvers provided to each examiner who participated in the study.**
(DOCX)

Checklist S1 **PRISMA Checklist.**
(DOC)
