# Supplementary figures and images for: Utility of the Physical Examination in Detecting Pulmonary Hypertension. A Mixed Methods Study
Source: PLoS One. 2014 Oct 24;9(10):e108499. doi: 10.1371/journal.pone.0108499 (PMC4208756; doi:10.1371/journal.pone.0108499)

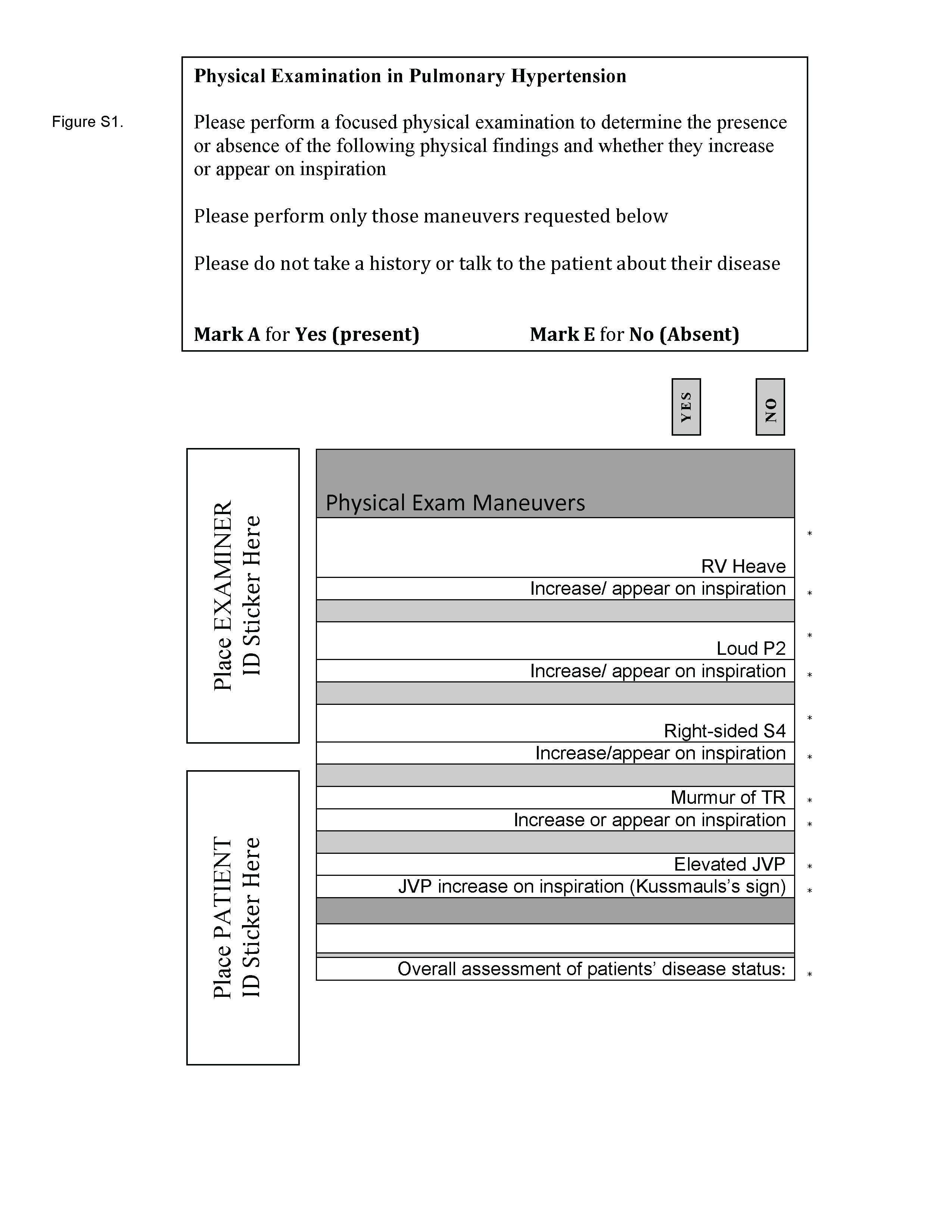

Supplement: Figure S1 — Standardized recording sheet used by examiners to record their findings for each patient. A Likert scale was used to estimate the examiner's impression regarding the likelihood of pulmonary hypertension at the end of each examination. (TIFF) [file pone.0108499.s001.tiff]
