# Supplementary material for: Utility of the Physical Examination in Detecting Pulmonary Hypertension. A Mixed Methods Study
Source: PLoS One. 2014 Oct 24;9(10):e108499. doi: 10.1371/journal.pone.0108499 (PMC4208756; doi:10.1371/journal.pone.0108499)
Supplement: Table S1 — Summary of the 31 studies were identified that assess the physical examination in pulmonary hypertension and were included in the final analysis. (DOCX) [file pone.0108499.s002.docx]

Table S1

| **Study** | **Design** | **Study population** | **Diagnostic Intervention Studied** | **PH diagnostic test** | **Proportion of patients with PH** |
| --- | --- | --- | --- | --- | --- |
| Bleifer et al. 1960[^16^](#_ENREF_16) | CS | VSD | Phonocardiography with simultaneous carotid artery pulse tracing (for timing the second heart sounds) | RHC | 7/44 |
| Chan et al. 2013[^7^](#_ENREF_7) | PC | PAH | Phonocardiography | RHC | 40/170 |
| Chen et al. 1996[^29^](#_ENREF_29) | CS | bioprosthetic AV or OM | Phonocardiography | 2D echo with doppler | 19/89 |
| Craige, 1959[^15^](#_ENREF_15)^+^ | CS | VSD | Phonocardiography, ecg | RHC | 13/35 |
| Gamboa et al., 1965[^30^](#_ENREF_30)^+^ | CS | VSD | Phonocardiography | RHC | 18/30 |
| Ghose et al. 1972[^31^](#_ENREF_31)^+^ | CaS | IPAH | Physical examination | RHC | 8/8 |
| Goryanina NK. 1964[^18^](#_ENREF_18) | No methods | AV stenosis and CHD | Phonocardiography | ? | ?/131 |
| Harris & Sutton, 1968[^25^](#_ENREF_25)^+^ | CS | Normal subjects | Phonocardiography | No measurement of PAP | 0/162 |
| Harris 1970[^21^](#_ENREF_21)^+^ | no methods | PH in CHD | no methods | No methods | 167/167 |
| Hollman et al. 1963 [^32^](#_ENREF_32) | CS | VSD | Phonocardiography | Pulmonary artery manometry at time of cardiac surgery | 40/93^#^ |
| Hubbard et al. 1957[^33^](#_ENREF_33)^+^ | RC | VSD | Physical examination | RHC | 39/50  (5/6 adults) |
| Kambe et al., 1978[^34^](#_ENREF_34) | CS | pulmonary regurgitation | Intrathoracic phonocardiography | RHC | 10/14 |
| Kapoor et al. 1968[^35^](#_ENREF_35) | CS | cor pulmonale | Physical examination | ? | 55/55 |
| Karnegis & Wang, 1964[^17^](#_ENREF_17) | CS | idiopathic PA dilatation | Phonocardiography, | RHC | 0/8 |
| Leatham & Gray 1956[^22^](#_ENREF_22) | CS | ASD; excluded severe pulmonary stenosis | Phonocardiography, auscultation | RHC | 10/40 |
| Leatham & Vogelpel, 1953[^36^](#_ENREF_36) | CS | patients with early systolic sounds | Phonocardiography, auscultation | RHC | 44/50 |
| Leo & Hultgren 1959[^37^](#_ENREF_37)^+^ | CS | mitral stenosis | Phonocardiography, auscultation | RHC or intraoperative measurements | 6/20 |
| Lessof 1959[^38^](#_ENREF_38)^+^ | CS | VSD | Phonocardiography, auscultation | RHC | ?/60 (?5 but does not indicate if there were more) |
| Longhini et al. 1991[^39^](#_ENREF_39) | CS | mitral stenosis | Phonocardiography with computer analysis | RHC | 6/30 |
| Lukomsky 1965[^40^](#_ENREF_40) | CS | mitral stenosis | Phonocardiography | RHC or measurement of pressure at time of mitral commisurotomy | 64/76* |
| Maciera-Coelho & Guimaraes. 1964[^41^](#_ENREF_41) | CS | ASD | Phonocardiography | RHC | 8/25 |
| Pilatis et al. 2000[^26^](#_ENREF_26) | RC | Liver transplant | Physical examination | RHC | 8/55 |
| Portaluppi et al. 1980[^42^](#_ENREF_42)^+^ | RC | acquired AS | Phonocardiography | RHC | 6/19* |
| Rich et al. 1987[^43^](#_ENREF_43)^+^ | CS?observational | primary pulmonary hypertension | Physical examination | RHC | 187/187 |
| Shaver et al. 1974[^20^](#_ENREF_20) | CS | mixed cardiac issues | Intracardiac phonocardiography | RHC | 9/22 |
| Slodki & Shah. 1965[^19^](#_ENREF_19) | CS | mitral valve disease or cor pulmonale | Phonocardiography | Not specified | 61/61 |
| Stein, Sabbah et al. 1980[^44^](#_ENREF_44) | CS | PH and controls | Intracardiac sound | RHC | 16/24 |
| Sutton et al. 1968[^24^](#_ENREF_24)^+^ | CS | PH secondary to CHD, PPH, group III PH | Phonocardiography | RHC | 116/150 |
| Ungerer et al. 1983[^28^](#_ENREF_28) | PC | PH secondary to systemic sclerosis | Physical examination | RHC | 16/49* |
| Wood 1952[^23^](#_ENREF_23) | RC | PH secondary to various causes | Physical examination | RHC | 44/44 |
| Xu et al., 2002[^8^](#_ENREF_8) | CS | mixed cardiac disease: (AS, AI, MS, MR, CAD, AMI, CHF, CM) | Phonocardiography | RHC | 10/23** |

+: study included children, CS: cross-sectional study, CaS: case series, PC: prospective cohort study, RC: retrospective cohort study, RHC: right heart catheterization, *:defined PH as mPAP ≥20mmHg, **:defined PH as sPAP >35mmHg

#: PH defined as mPAP ≥30% of systemic pressure as described by authors and in absence of additional information
