# Supplementary material for: Utility of the Physical Examination in Detecting Pulmonary Hypertension. A Mixed Methods Study
Source: PLoS One. 2014 Oct 24;9(10):e108499. doi: 10.1371/journal.pone.0108499 (PMC4208756; doi:10.1371/journal.pone.0108499)
Supplement: Text S1 — Details of the physical examination maneuvers provided to each examiner who participated in the study. (DOCX) [file pone.0108499.s003.docx]

Text S1.

**Physical examination techniques used during an evaluation for pulmonary hypertension:**

*Loud P2:*

Auscultate over the base of the heart during inspiration, when splitting of the second heart sound is maximal. If the second component of the second heart sound is louder than the first, then the patient is said to have a loud P2.

*RV Enlargement:*

With the patient supine and chest exposed, place the ulnar edge of the palm on the left parasternal border touching the 3^rd^, 4^th^ and 5^th^ interspaces. Feel for a systolic tap (heave).

*Right sided S4:*

Right sided S4 is best heard over the left sternal border near the xiphoid if originating from the right ventricle. The S4 is a low-pitched sound preceding the first heart sound (S1) and is preferentially heard with the bell of the stethoscope. Such a detected sound that becomes louder on inspiration is indicative of right sided origin

*Tricuspid regurgitation:*

The murmur of tricuspid regurgitation (TR) is a systolic murmur best heard at the left sternal border in the fourth and fifth intercostal spaces. In contrast to the murmur of mitral regurgitation, the TR murmur becomes louder with inspiration and does not radiate to the axilla. To distinguish the murmur of TR from one originating from a semi-lunar valve, the TR murmur should begin with S1 rather than after the short delay that is manifest in association with an aortic or pulmonic ejection murmur that represents the silent pre-ejection period of systole after the atrioventricular valves close.

*Jugular venous pressure/Kussmaul sign:*

Begin with the patient relaxing comfortably on the examination table with the head of the bed elevated between 30 and 45 degrees. Inspect for the height of pulsations in the right internal jugular vein (posterior and superior to the medial fourth of the clavicle) in quiet inspiration and expiration. To find the top of the column, it may be necessary to adjust the elevation of the head of the bed. Measure the vertical distance between the top of the column and the sternal angle. The JVP is considered abnormally elevated if this distance is greater than 4.5 cm. Under normal circumstances, the jugular venous pressure should decrease with inspiration. A positive Kussmaul sign is a rise in jugular pressure during inspiration.
